# Combined analysis and validation for DNA methylation and gene expression profiles associated with prostate cancer

**DOI:** 10.1186/s12935-019-0753-x

**Published:** 2019-03-04

**Authors:** Yanqiu Tong, Yang Song, Shixiong Deng

**Affiliations:** 1 Laboratory of Forensic Medicine and Biomedical Informatics, Chongqing Medical University, Chongqing, 400016 People’s Republic of China; 2 School of Humanity, Chongqing Jiaotong University, Chongqing, 400074 People’s Republic of China; 3 Department of Device, Chongqing Medical University, Chongqing, 400016 People’s Republic of China

**Keywords:** Bioinformatics, Prostate cancer, Differentially expressed gene, DNA methylation

## Abstract

**Background:**

Prostate cancer (PCa) is a malignancy cause of cancer deaths and frequently diagnosed in male. This study aimed to identify tumor suppressor genes, hub genes and their pathways by combined bioinformatics analysis.

**Methods:**

A combined analysis method was used for two types of microarray datasets (DNA methylation and gene expression profiles) from the Gene Expression Omnibus (GEO). Differentially methylated genes (DMGs) were identified by the R package minfi and differentially expressed genes (DEGs) were screened out via the R package limma. A total of 4451 DMGs and 1509 DEGs, identified with nine overlaps between DMGs, DEGs and tumor suppressor genes, were screened for candidate tumor suppressor genes. All these nine candidate tumor suppressor genes were validated by TCGA (The Cancer Genome Atlas) database and Oncomine database. And then, the gene ontology (GO) and Kyoto Encyclopedia of Genes and Genomes pathway (KEGG) enrichment analyses were performed by DAVID (Database for Annotation, Visualization and Integrated Discovery) database. Protein–protein interaction (PPI) network was constructed by STRING and visualized in Cytoscape. At last, Kaplan–Meier analysis was performed to validate these genes.

**Results:**

The candidate tumor suppressor genes were IKZF1, PPM1A, FBP1, SMCHD1, ALPL, CASP5, PYHIN1, DAPK1 and CASP8. By validation in TCGA database, PPM1A, DAPK1, FBP1, PYHIN1, ALPL and SMCHD1 were significant. The hub genes were FGFR1, FGF13 and CCND1. These hub genes were identified from the PPI network, and sub-networks revealed by these genes were involved in significant pathways.

**Conclusion:**

In summary, the study indicated that the combined analysis for identifying target genes with PCa by bioinformatics tools promote our understanding of the molecular mechanisms and underlying the development of PCa. And the hub genes might serve as molecular targets and diagnostic biomarkers for precise diagnosis and treatment of PCa.

**Electronic supplementary material:**

The online version of this article (10.1186/s12935-019-0753-x) contains supplementary material, which is available to authorized users.

## Background

DNA methylation, one of the most important epigenetic factors, has been studied extensively over several decades, and its influence in a variety of human diseases, most malignancy tumor, diagnostic biomarkers and therapeutic targets, has been firmly measured and evaluated [1–6]. Recently epigenetic study has identified that DNA methylation is an important biological mechanism for tumor occur and development [5]. In CpG islands, aberrant methylation could influence the functions of tumor suppressor genes by altering their expression levels. CpG islands are located in or near promoter regions of the genome, aberrant methylation genes in CpG islands are often hypermethylated and may cause silencing of tumor suppressor genes [7]. Variations of DNA methylation exist at CpG islands, including gene hypermethylation-low regulation and hypomethylation-high regulation.

Several studies that have done to investigate DNA methylation in gene body has positively correlated with gene expression by increasing transcription activity [8]. This may be caused by blocking the intragenic promoter activity or affecting the methylation status of repetitive sequence within the transcription unit [9]. So DNA methylation in gene body may be an interesting additional therapy target for cancer diagnosing and treatment.

Prostate cancer (PCa) is the second most frequently diagnosed male-specific malignancy tumor in western countries. According to the World Health Organization’s International Agency for Research on Cancer, 1.1 million men were diagnosed with prostate cancer worldwide in 2012, accounting for 15% of all cancer diagnosed in men [6]. PCa is considered as a heterogeneous disease [10]. Accumulating evidence has also demonstrated that multiple genes and cellular pathways participate together in the occurrence and development of PCa. Tumor led by epigenetic mutation through cells may grow and reproduce uncontrollably [11]. Hypomethylation of CpG can cause chromosome instability [12]. Illumina Infinium 450 k microarray and DNA microarray have utilized to investigate DNA methylation and gene expression in molecular mechanism, biological process, molecular diagnosis, tumor molecular, biomarker and drug targets discovery [13–15].

Many gene expression profiling analysis and aberrant methylation analysis were introduced for differentially expressed genes (DEGs) and differentially methylated genes (DMGs) [16]. However, separated analysis of DEGs and DMGs are limited [17, 18]. So it is necessary to make jointly analyze for both gene expression profiling microarray and gene methylation profiling microarray in PCa. In this study, gene methylation profiling datasets and gene expression profiling datasets were analyzed by bioinformatics tools for screening the DMGs and DEGs. Later, the overlapping of hypermethylation genes, down-regulated genes and tumor suppressor genes were used to identify the candidate tumor suppressor genes. For validating these candidate tumor suppressor genes, TCGA database was used to identify the CpG islands and Oncomine database was used to validate the analyzing result. At last, the biological functions and pathways analyzing were discussed for the molecular mechanism.

## Methods

### Datasets

In this study, the gene methylation profiling datasets and gene expression profiling datasets were downloaded from Gene Expression Omnibus (https://www.ncbi.nlm.nih.gov/geo) database. All these gene methylation profiling datasets were based on GPL13534 platform (Illumina HumanMethylation450 BeadChip). For the gene expression profiling datasets were based on GPL570 platform (Affymetrix Human Genome U133 Plus 2.0 Array). For each dataset, only the samples associated with PCa were selected (Table 1).

**Table 1:** Datasets for gene methylation profiling and gene expression profiling associated with PCa

Dataset	Platform	Tumor tissue samples	Normal tissue samples
Gene methylation profiles			
GSE52955	GPL13534	25	5
GSE73549	GPL13534	57	15
GSE76938	GPL13534	73	63
GSE84749	GPL13534	20	4
Gene expression profiles			
GSE26910	GPL570	6	6
GSE30174	GPL570	70	10
GSE46602	GPL570	36	14
GSE55945	GPL570	13	8
GSE69223	GPL570	15	15

### Data preprocessing and analyzing

R package affy was used to explore oligonucleotide array analysis by the robust multiarray average (RMA) algorithm [19]. While R package methylumiIlluminaHumanMethylation450kmanifest, limma, minfi, watermelon and IlluminaHumanMethylation450kanno.ilmn12.hg19 were used to analyze the gene methylation profiling data and gene expression profiling data in order to identify DMGs and DEGs. And the R package limma in RStudio 1.1.453 was installed to identify genes that were differentially expressed between normal and tumor samples. The Benjamini and Hochberg (BH) procedure were obtained to control the False Discovery Rate (FDR) [20]. Then the log_2_-fold change (log_2_FC) was calculated. The adjusted *P* value < 0.05 and |log2FC| > 2.0 were considered as the cutoff value for DMGs and DEGs screening.

### Functional and pathway enrichment analysis of DMGs and DEGs

In order to analyze the DMGs and DEGs for the functional enrichment, GO enrichment and KEGG pathway analysis were performed using DAVID (https://david.ncifcrf.gov). In this paper, DMGs and DEGs were uploaded to online analysis tool to systematically investigate biological meanings behind these genes [21]. Gene ontology analysis (GO) is a common useful method for annotating genes for identifying biological process (BP), cellular component (CC) and molecular function (MF) [22, 23]. At the same time, Kyoto Encyclopedia of Genomes (http://www.genome.jp) pathway enrichment analysis was conducted for candidate genes and prostate specific antigen (PSA) [24].

### Protein–protein interaction (PPI) network construction and module analysis

STRING database (https://string-db.org) was used for protein–protein interaction (PPI) analysis in order to investigate the molecular mechanisms. STRING database (version 10.5) covers 9,643,763 proteins from 2031 organisms. While network analysis is a useful method for uncovering all kinds of protein–protein interactions networks. It can measure networks by nodes, edges, degrees and network structures, so it can help us to identify hub genes and key protein community. Then, PPI networks were constructed by Cytoscape software (http://www.cytoscape.org). Cytoscape is an open source software platform for visualizing molecular interaction networks, biological pathways and integrating these networks with annotations, gene expression profiles and other state data. A plugin named cytoHubba was introduced to screen hub genes of PPI network in Cytoscape. CytoHubba can provide 12 topological analysis methods including Betweenness, BottleNeck, Closeness, Clustering Coefficient, Degree, DMNC, EcCentricity, EPC, MCC, MNC, Radiality and Stress based on shortest paths [25].

## Results

### Normalization of gene methylation profiling and gene expression profiling

**Fig. 1:**
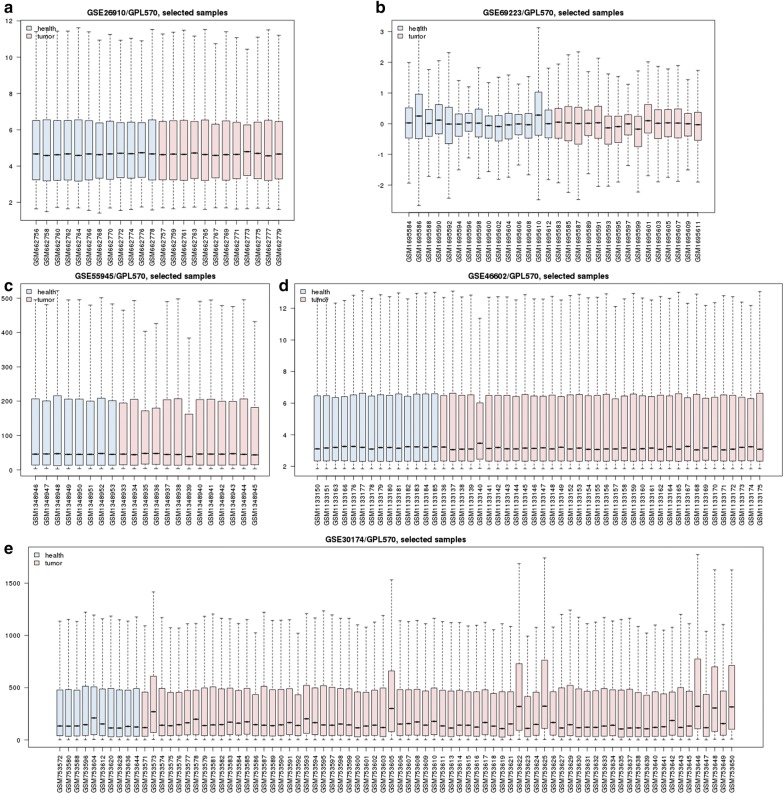
Data distribution of gene expression profiling data (**a** GSE26910; **b** GSE69223; **c** GSE55945; **d** GSE46602; **e** GSE30174)

**Fig. 2:**
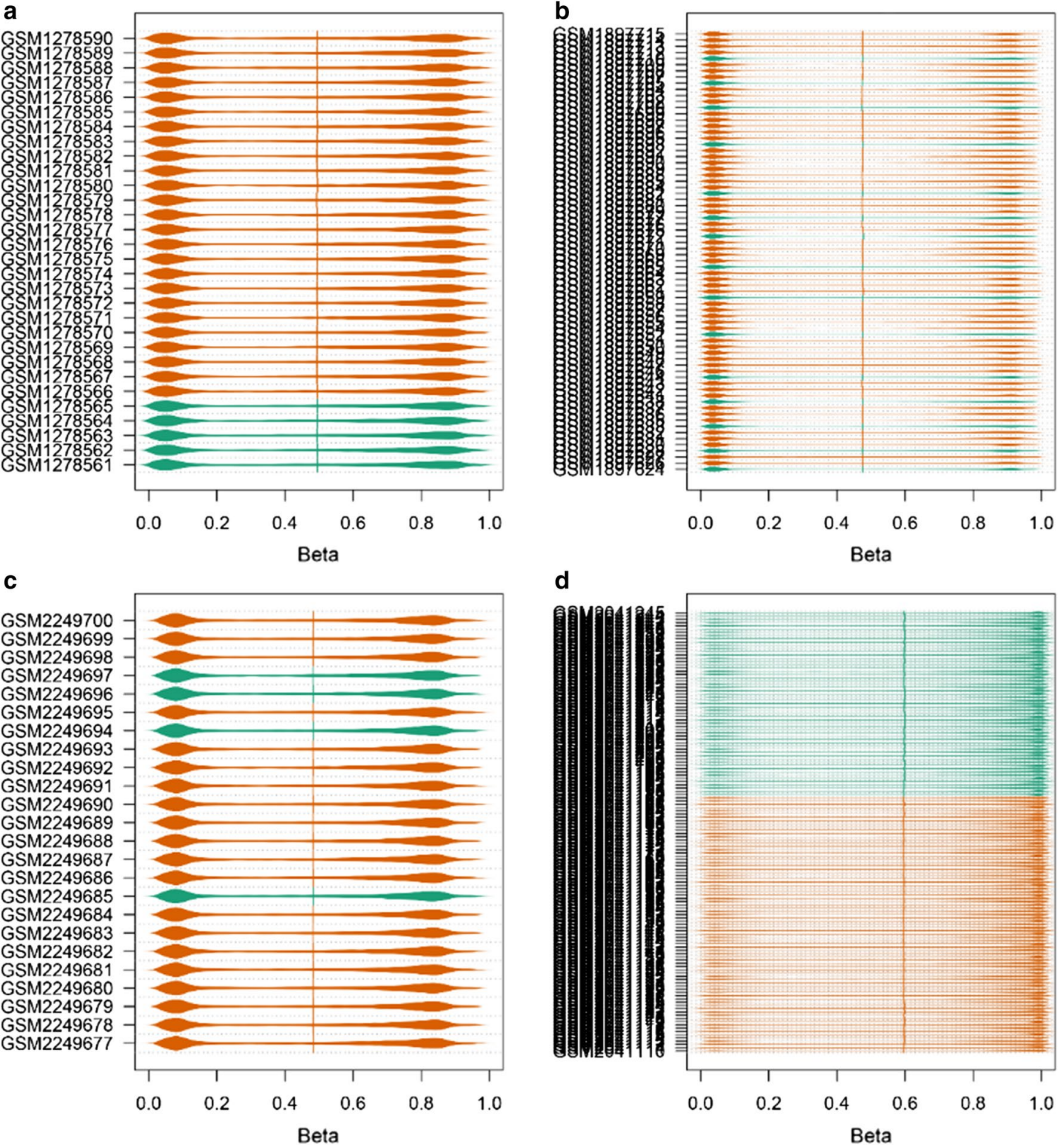
Data distribution of methylation profiling data (**a** GSE52955; **b** GSE73549; **c** GSE84749; **d** GSE76938)

In this study, the five gene expression profiling dataset (Fig. 1) and four gene methylation profiling dataset (Fig. 2) were separately analyzed by R package affy and online GEO2R (https://www.ncbi.nlm.nih.gov/geo/geo2r/) for screening DEGs and DMGs. All the gene expression profiling microarray chips were based on affymetrix GPL 570 platform. On affymetrix arrays, genes are represented by one or more probe sets, which are short oligonucleotides covering distinct sections of the gene synthesised in place through photolithography [26].

While on Illumina BeadChips arrays, 50 base pairs Infinium methylation probes synthesised by bisulfite conversion of unmethylated cytosines, which are randomly dispersed over the array [27]. This random allocation means that each probes was represented a random number of times on each array.

**Fig. 3:**
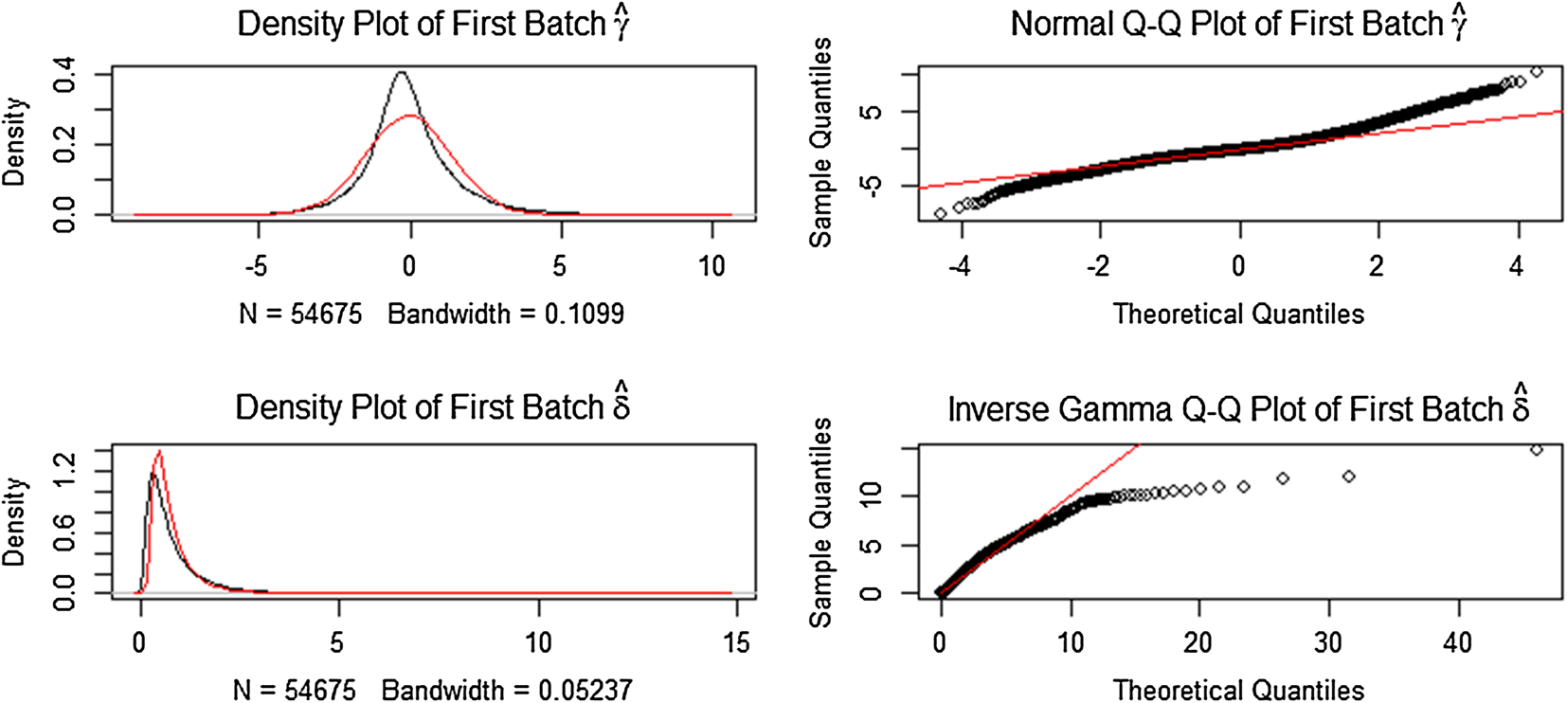
Reject batch effects for gene expression profiling data

**Fig. 4:**
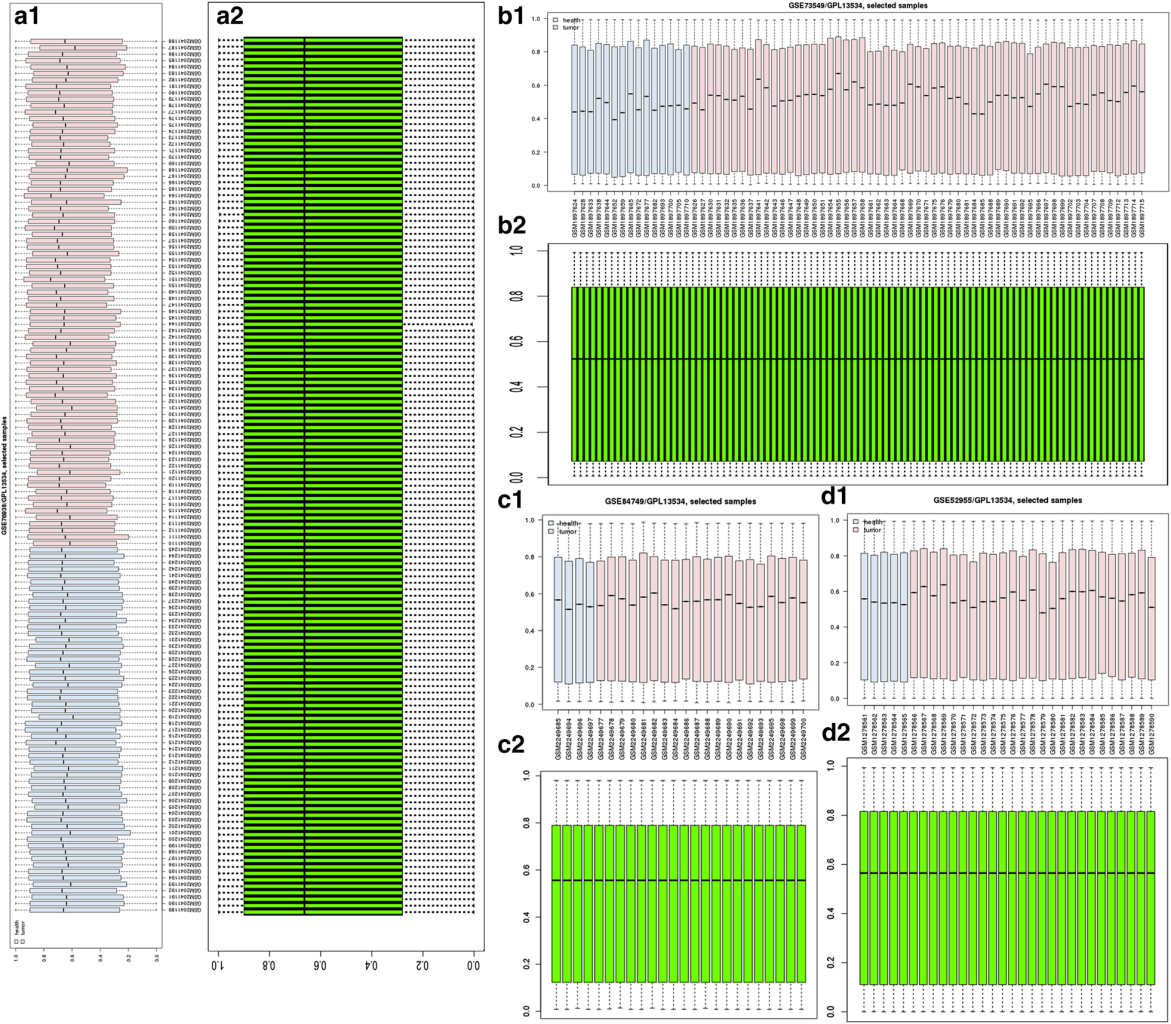
Normalization of gene methylation profiling (**a1** GSE76938 gene methylation profiling data value distribution; **a2** normalization of GSE76938 gene methylation profiling data; **b1** GSE73549 gene methylation profiling data value distribution; **b2** normalization of GSE73549 gene methylation profiling data; **c1** GSE84749 gene methylation profiling data value distribution; **c2** normalization of GSE84749 gene methylation profiling data; **d1** GSE52955 gene methylation profiling data value distribution; **d2** normalization of GSE52955 gene methylation profiling data)

Microarrays and bead chips technologies depend on a complicated set of reagents and hardware, along with highly trained personnel, to produce accurate measurements. Both biological and non-biological factors will affect the results during the experiment when a series of complicated set of reagents and hardware varied [28]. So batch effects in different microarrays and bead chips should be focused after high-throughput experiments. Batch effects may occur at different laboratories, seasons and days. In order to eliminate batch effect problem, the surrogate variable analysis was conducted to reduce the batch effects depend on R package sva (Fig. 3). The sva package contains functions for removing batch effects and other unwanted variation in high-throughput experiment. The value distribution of methylation profiling data and the normalization of gene methylation profiling data were shown in Fig. 4.

### Identification of DEGs and DMGs in PCa

**Fig. 5:**
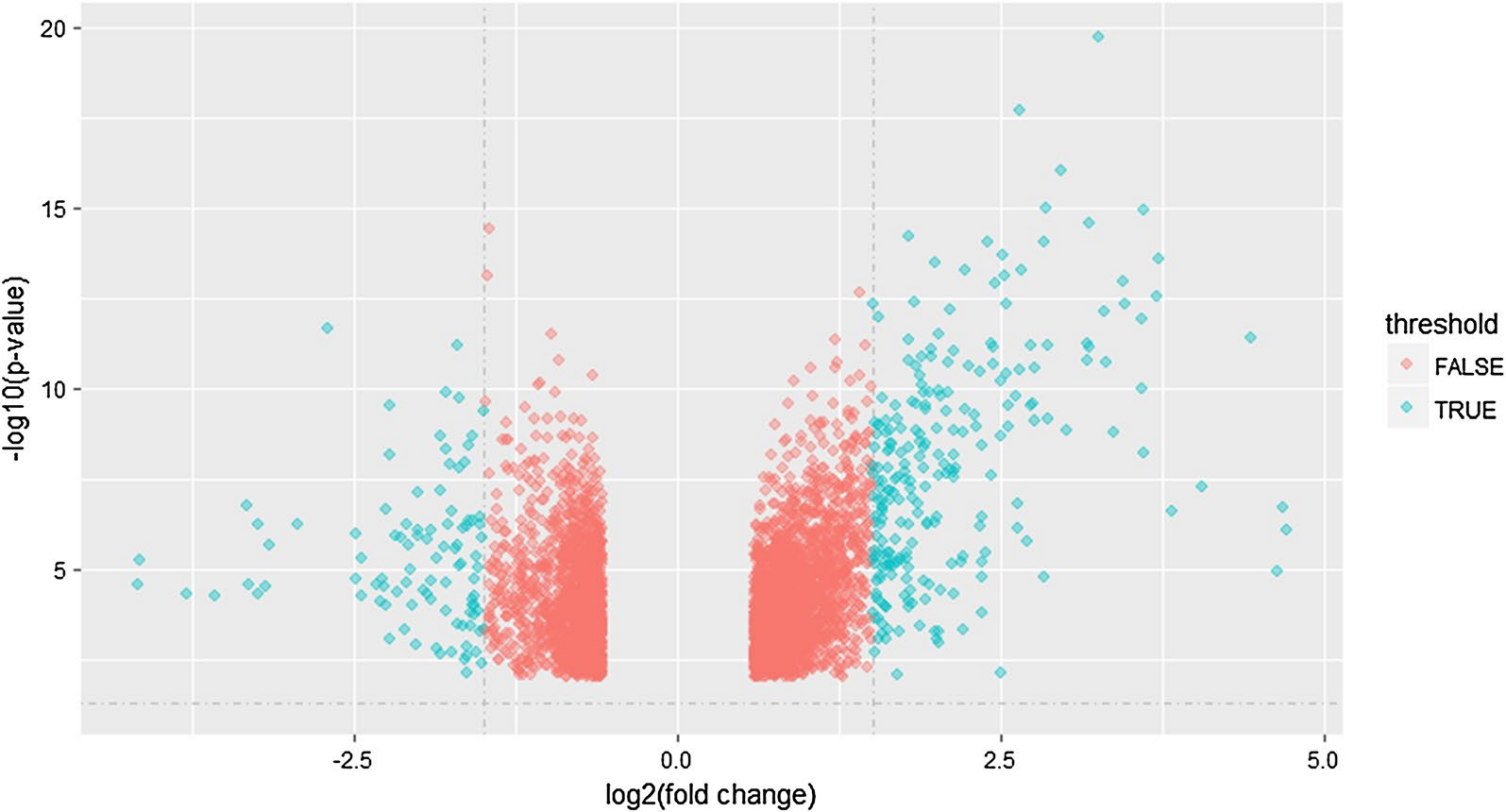
Volcano plot of DEGs in gene expression datasets

**Fig. 6:**
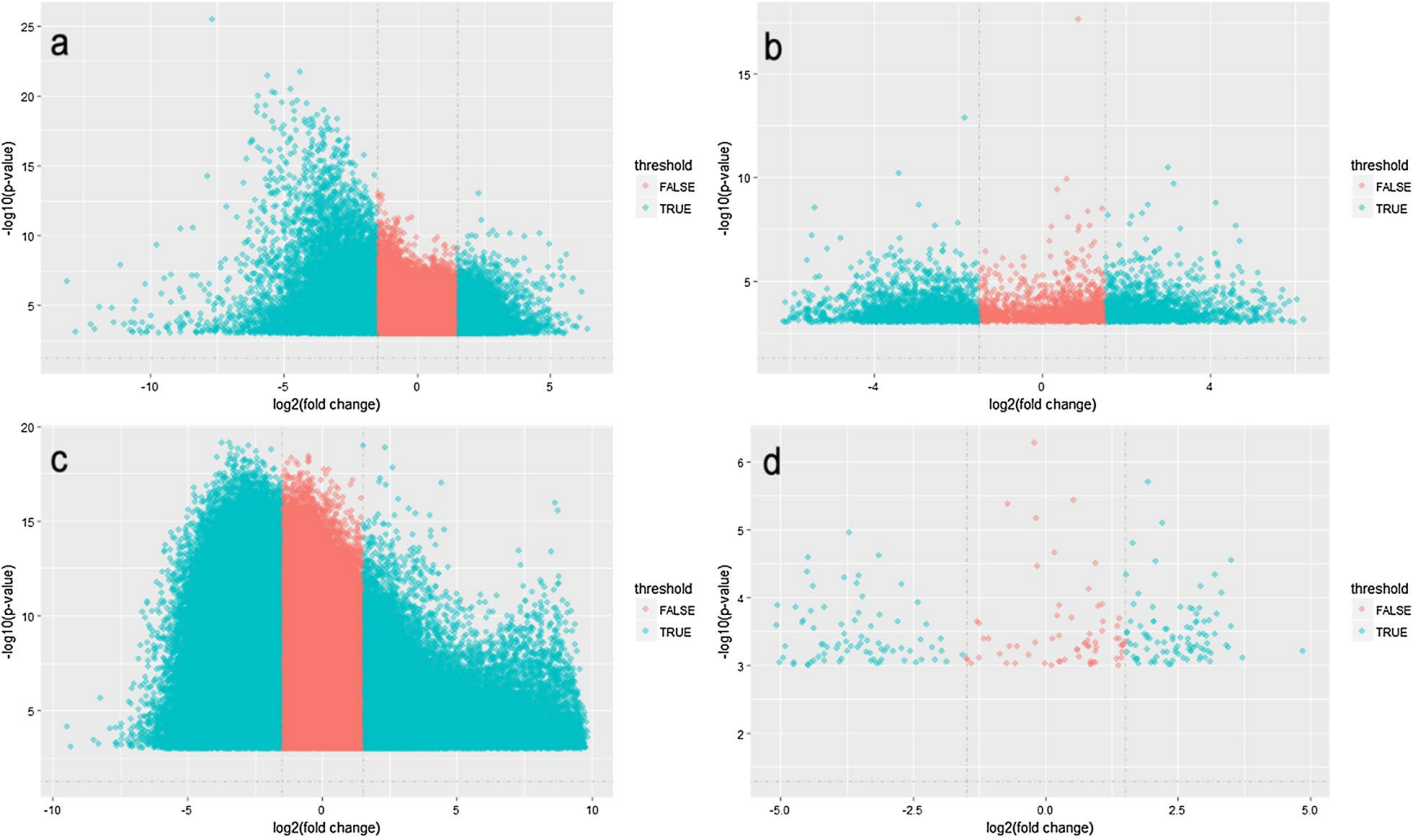
Volcano plot of DMGs in gene methylation datasets (**a** GSE52955; **b** GSE73549; **c** GSE76938; **d** GSE84749)

After data normalizing and removing batch effects, total of 1331 DEGs were identified, using P < 0.05 and log_2_-fold change |log2FC| > 2.0 criteria, of which, 938 up-regulated genes and 393 down-regulated genes (Fig. 5). For DMGs, total of 3261 DMGs were identified according P < 0.05, log_2_-fold change |log2FC| > 2.0 and fwer < 0.5 criteria, of which, 2699 hypermethylation genes and 562 hypomethylation genes. The result has been shown as the volcano plot in Fig. 6.

### Aberrantly methylated-differentially expressed genes in PCa

**Fig. 7:**
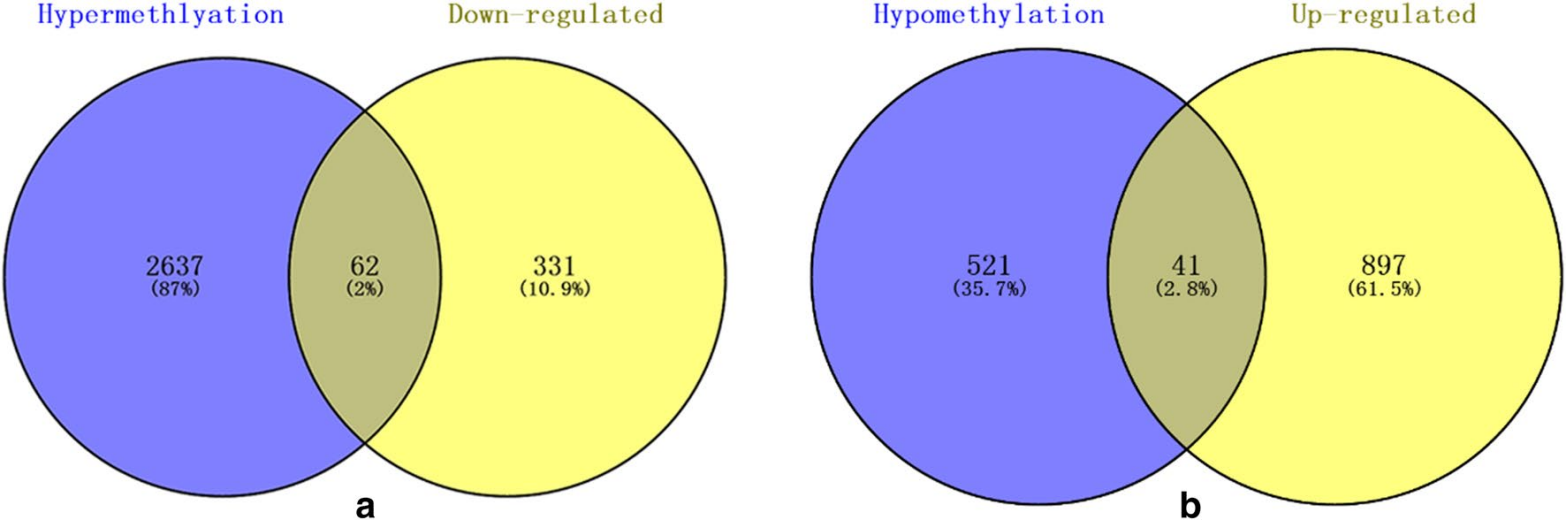
Venn diagram [29] for aberrantly methylated-differentially expressed genes by overlapping gene expression datasets and gene methylation datasets (**a** hypermethylation and down-regulated genes; **b** hypomethylation and up-regulated genes)

Then, totally 62 hypermethylation-low genes were obtained by overlapping 2699 hypermethylation genes and 393 down-regulated genes in Fig. 7. And 41 hypomethylation-high genes were obtained by overlapping 362 hypomethylation genes and 938 up-regulated genes in Fig. 7.

### Integrating dataset for screening candidate tumor suppressor genes

**Fig. 8:**
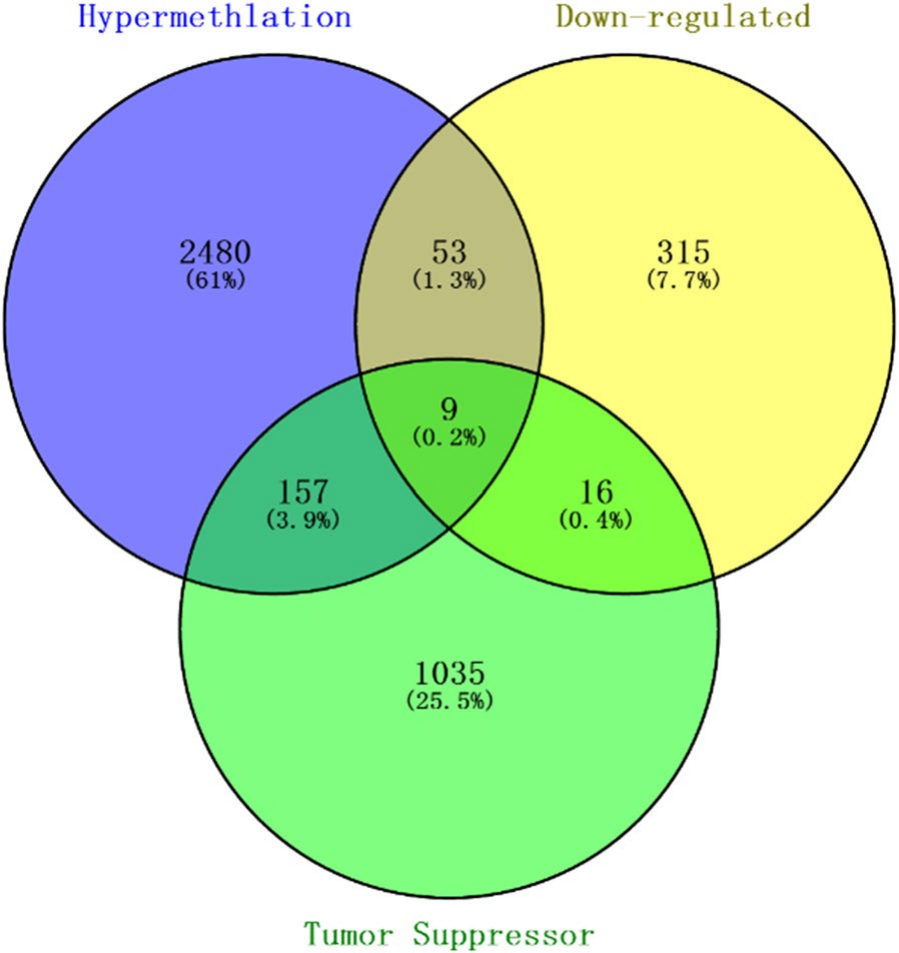
Venn diagram [29] for candidate tumor suppressor genes by overlapping hypermethylation genes, down-regulated genes and tumor suppressor genes

Then, totally 9 candidate tumor suppressor genes were obtained by overlapping 2699 hypermethylation genes, 393 down-regulated genes and 1217 human tumor suppressor genes [30] in Fig. 8.

**Fig. 9:**
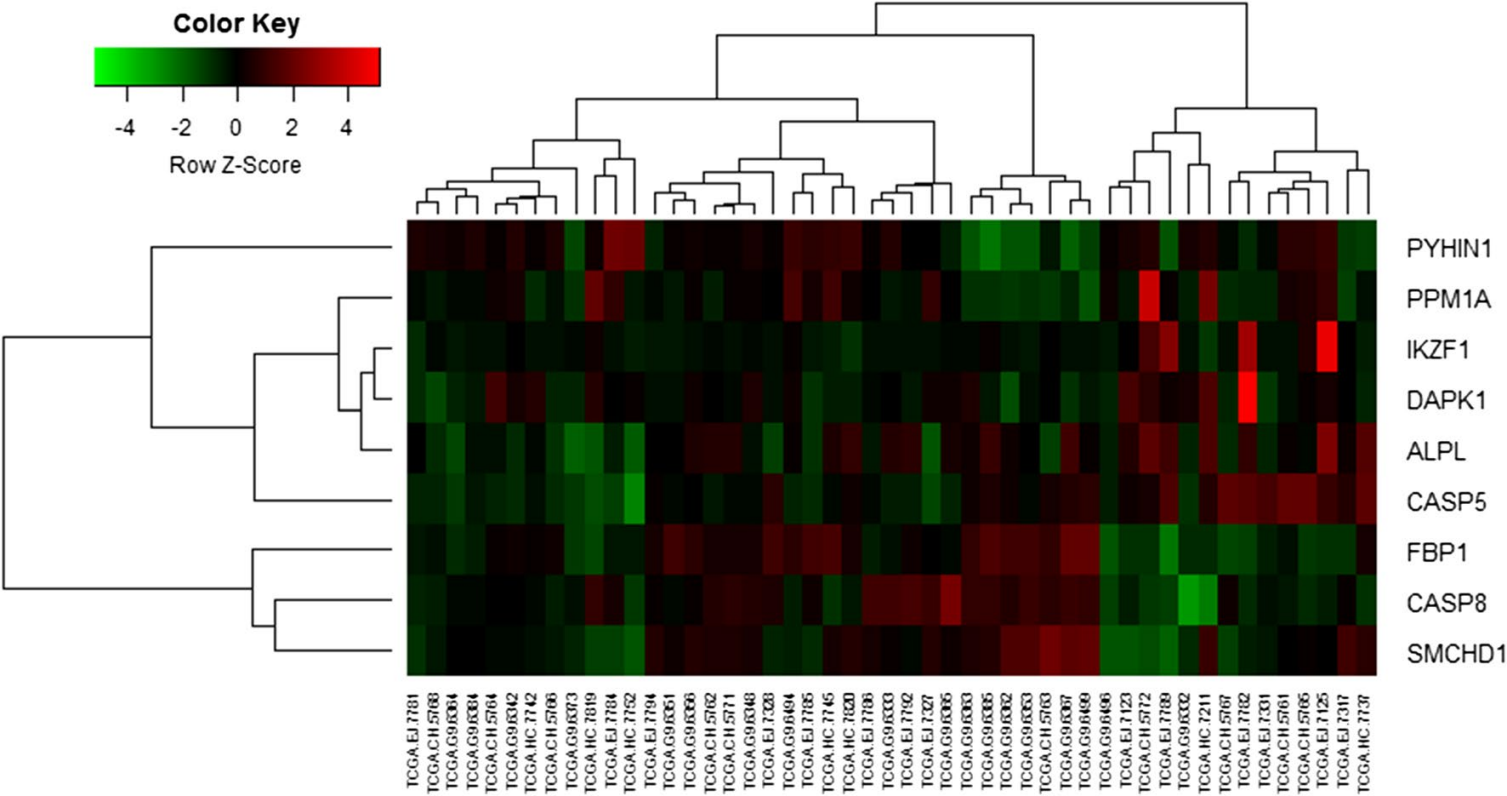
Nine candidate tumor suppressor genes

The heat map of nine candidate tumor suppressor genes (IKZF1, PPM1A, FBP1, SMCHD1, ALPL, CASP5, PYHIN1, DAPK1 and CASP8) was shown as Fig. 9. Although these tumor suppressor genes were hypermethylation-low genes, these genes were not significant in some samples for TCGA database.

### Validation of candidate tumor suppressor genes in TCGA database

**Fig. 10:**
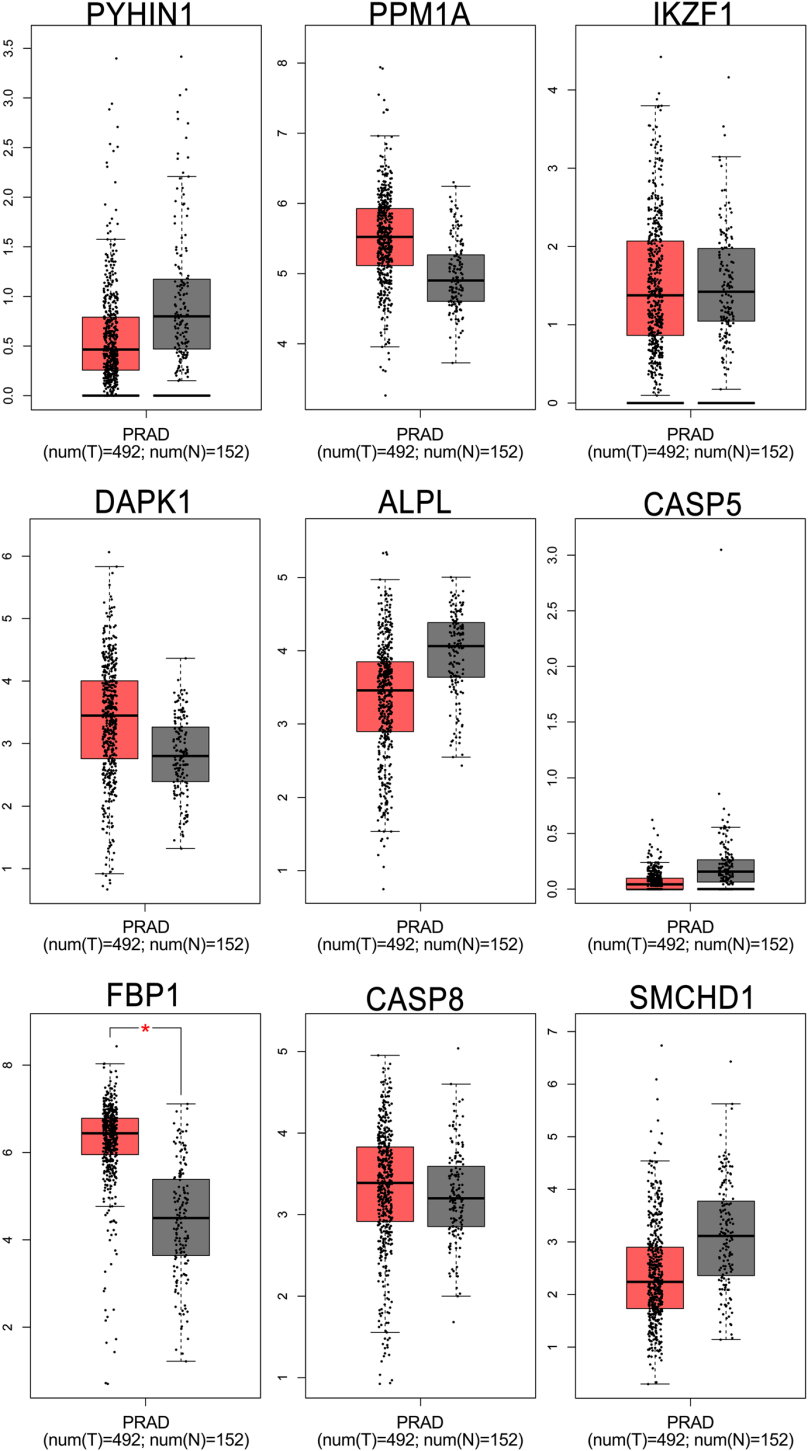
PCa-related candidate tumor suppressor genes expression in TCGA database

**Fig. 11:**
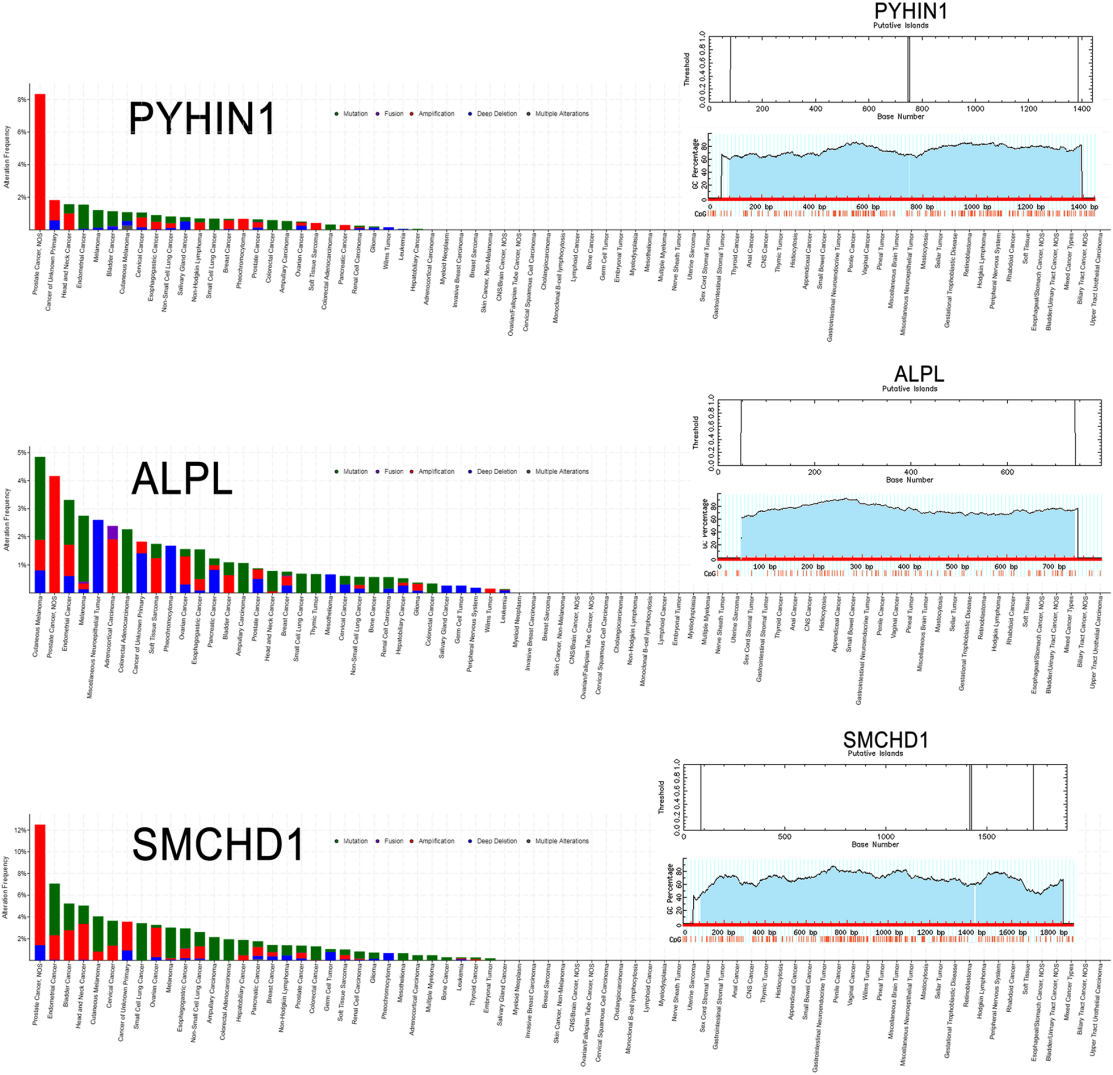
Pan-cancer analysis of PCa-related candidate tumor suppressor genes in normal samples

**Fig. 12:**
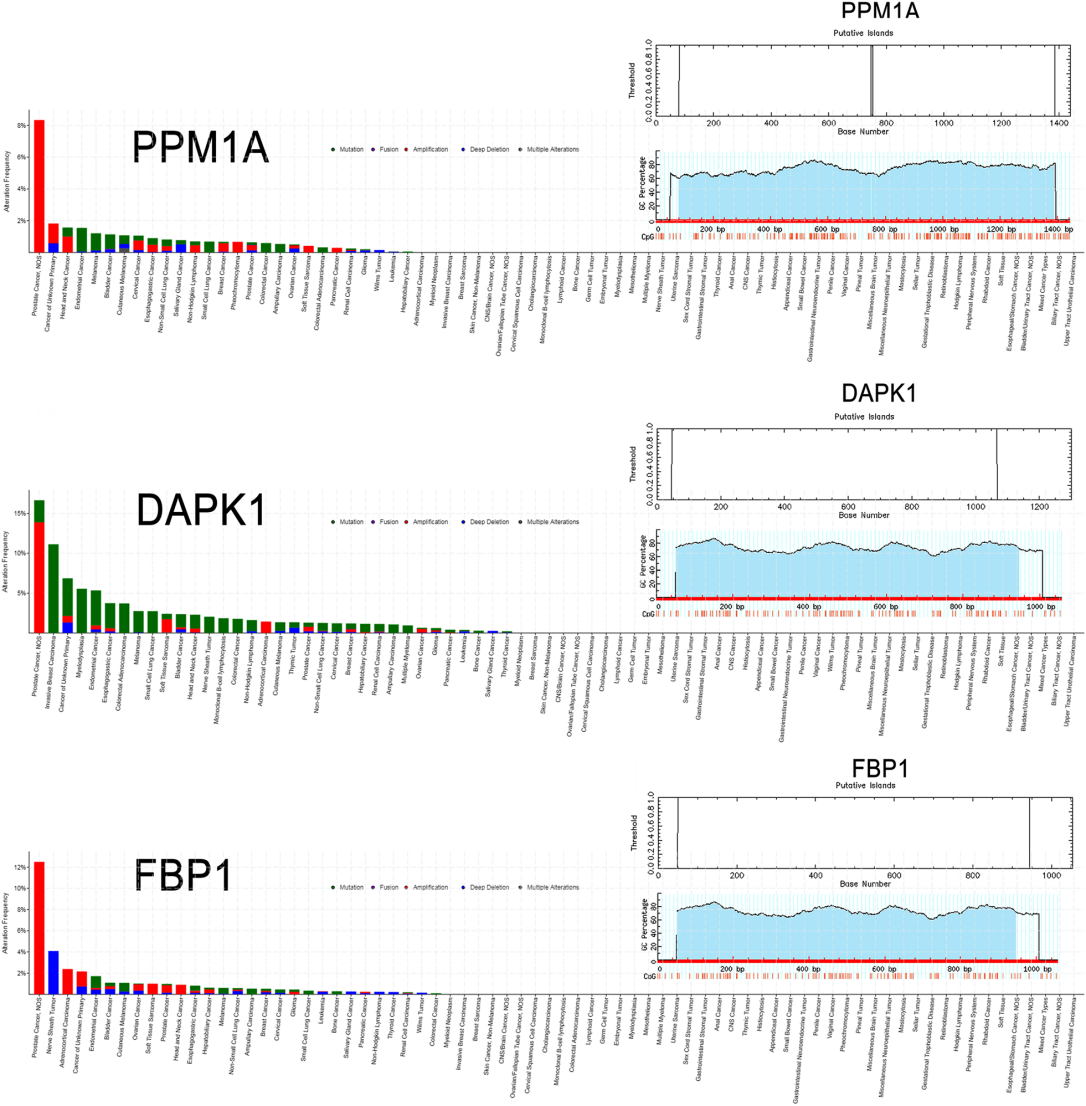
Pan-cancer analysis of PCa-related candidate tumor suppressor genes in tumor samples

To further investigate the candidate tumor suppressor genes, TCGA database was used to validate these results. The outcome was shown as Fig. 10. The candidate tumor suppressor genes were separately significant in tumor group and normal group except IKZF1, CASP5 and CASP8, which significantly indicate that PPM1A, DAPK1 and FBP1 were hypermethylation in tumor samples, at the same time, PYH1N1, ALPL and SMCHD1 were hypermethylation in normal samples. And then, in order to further confirm these candidate tumor suppressor genes the MethPrimer [31] and cpgplot software were used to predict the CpG islands in Figs. 11 and 12. At the same time, pan-cancer analysis also shows that PPM1A, DAPK1, FBP1, PYHIN1, ALPL and SMCHD1 have significant amplification in PCa.

### Integrated Oncomine database and survival analysis for candidate tumor suppressor genes

**Fig. 13:**
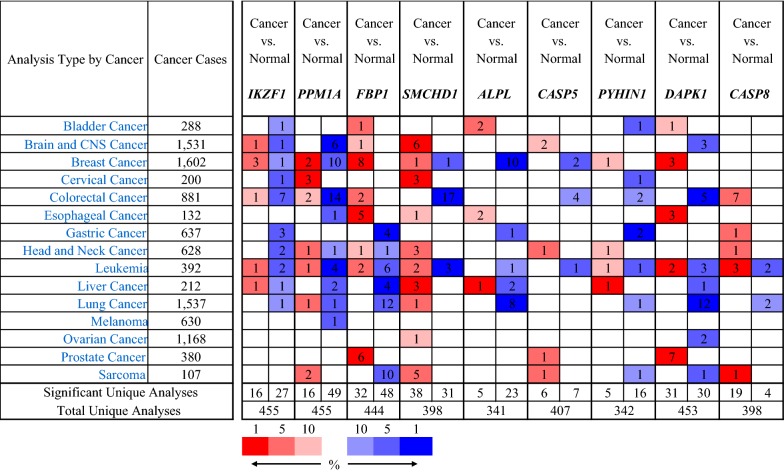
The landscape of the candidate tumor suppressor genes in human cancers (the more intense red indicates over-expression; the more intense blue indicates under-expressed gene.)

To confirm the candidate tumor suppressor genes expression between tumor and normal tissues in multiple cancers, the Oncomine database was performed to analyze the different expression. Using P < 0.01 and |log2FC| > 1.5 criteria, a total of 455, 455, 444, 398, 341, 407, 342, 453 and 398 unique analyses for IKZF1, PPM1A, FBP1, SMCHD1, ALPL, CASP5, PYHIN1, DAPK1 and CASP8 were shown in Fig. 13. In 80 studies, FBP1 was ranked by the top 10% of gene rank indicating significant statistical differences, 6 of which revealed higher expression level in tumor than normal tissues. For higher expression of CASP5, there was only one dataset listed. Up-regulated KAPK1 was founded in cancers based on sixty-one significant analyses.

**Fig. 14:**
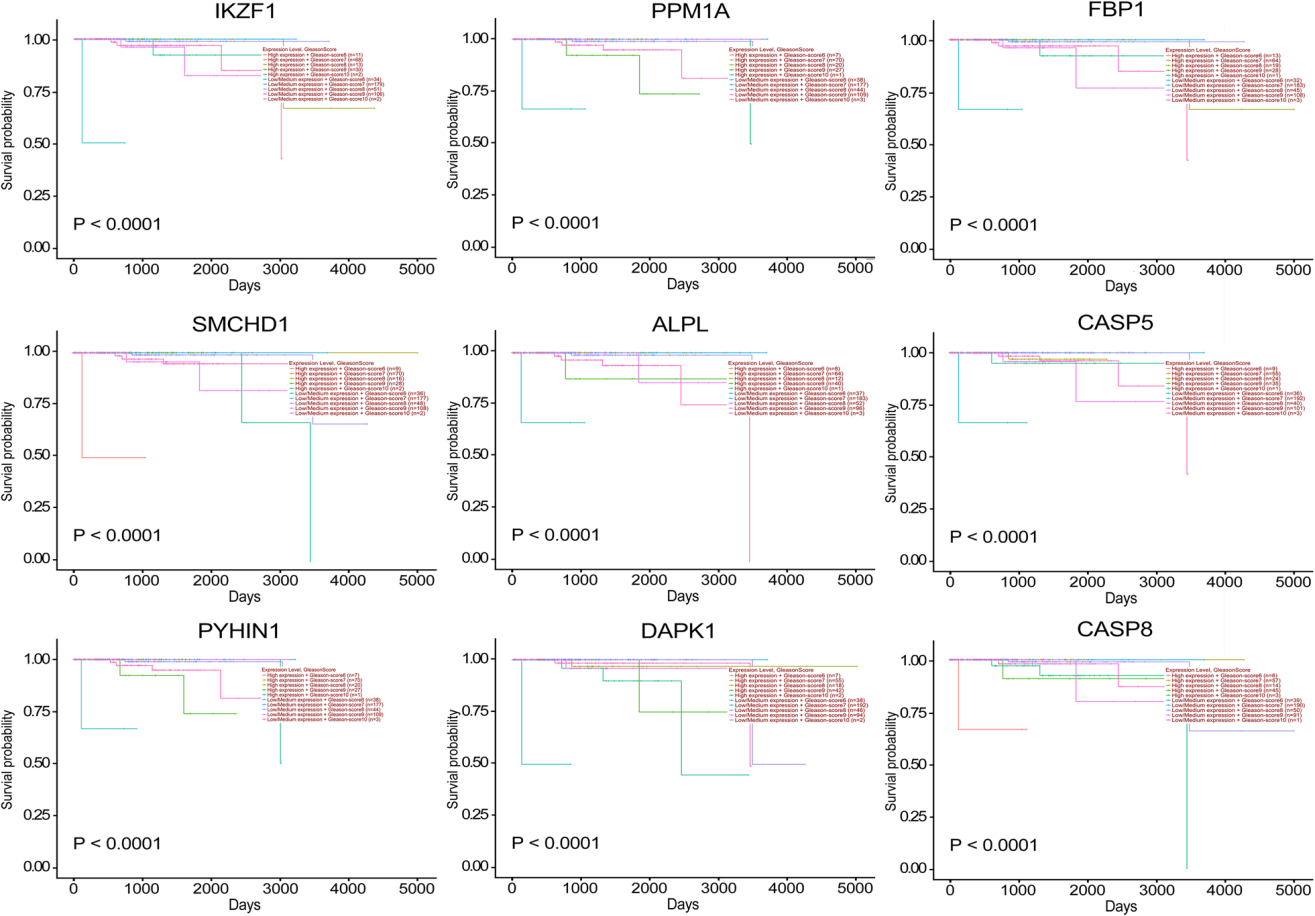
Kaplan-Meier survival analysis of candidate tumor suppressor genes associated with PCa [32]

To further confirm the candidate tumor suppressor genes, Kaplan–Meier analysis was performed in Fig. 14. All the low expression values of tumor suppressor genes are all significantly associated with poor prognosis while a high expression of tumor suppressor genes are associated with good prognosis.

**Fig. 15:**
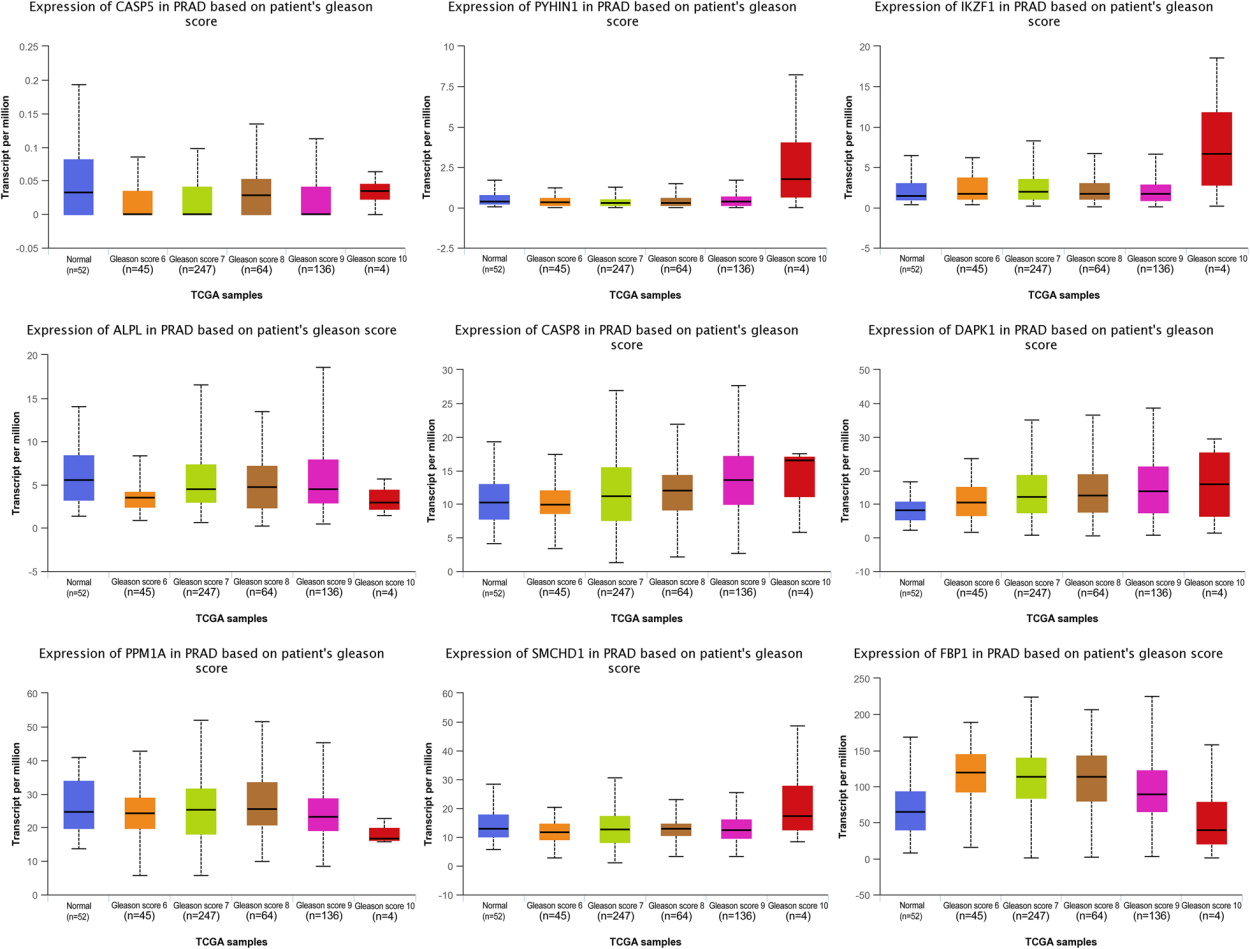
Expression of candidate tumor suppressor genes according Gleason Score system

The Gleason Score (GS) system for grading PCa is a standard evaluation method that has different stratification: GS ≤ 6, 3 + 4, 4 + 3, 8, 4 + 5, 5 + 4, 10, respectively to Gleason Grading Group 1, 2, 3, 4, and 5 [33]. Expression of candidate tumor suppressor genes according Gleason Score system is shown in Fig. 15. A low Gleason score (≤ 6) indicates good prognosis without risk of lymphatic metastasis whereas a high Gleason score (> 8) is associated with distal metastasis.

The expression of IKZF1, PPM1A, FBP1, SMCHD1, ALPL, CASP5, PYHIN1, DAPK1 and CASP8 candidate tumor suppressor genes was significantly deregulated in PCa by Gleason Score (Table 2). The significant genes observed were PYHIN1, IKZF1, CASP8, DAPK1 and SMCHD1 expression in tumor samples. Furthermore, FBP1 was deregulated at Gleason Score 6–9.

**Table 2:** Expression of candidate tumor suppressor genes in different PCa stage

Candidate tumor suppressor gene	Gleason Score (GS)				
	GS = 6	GS = 7	GS = 8	GS = 9	GS = 10
IKZF1	No sig	No sig	No sig	No sig	Up
PPM1A	No sig	No sig	No sig	Down	Down
FBP1	Up	Up	Up	Up	Down
SMCHD1	No sig	Down	No sig	No sig	Up
ALPL	Down	Down	Down	Down	Down
CASP5	Down	Down	No sig	Down	No sig
PYHIN1	No sig	No sig	No sig	No sig	Up
DAPK1	Up	Up	Up	Up	Up
CASP8	No sig	Up	Up	Up	Up

Up: up-regulated; down: down-regulated; no sig: no significant

### Gene ontology enrichment analysis and KEGG pathway analysis

In order to uncover these genes affected by altered DNA methylation status, all hypermethylation-low expression genes and hypomethylation-high expression genes were uploaded to DAVID database to identify overrepresented BP categories and KEGG pathways. The top 10 significant GO enrichments of biological processes were illustrated in Table 3.

**Table 3:** GO terms analysis of aberrantly methylated-differentially expressed genes in PCa

Category	Term	P-value
Hypermethylation-low expression	Cytokine-mediated signaling pathway	1.38E−04
	Benzene-containing compound metabolic process Kynurenine metabolic process	3.36E−04
	Positive regulation of cysteine-type endopeptidase activity involved in apoptotic process	3.36E−04
	Indolalkylamine catabolic process	3.78E−04
	Response to vitamin	4.19E−04
	Cellular response to mechanical stimulus	4.19E−04
	Tryptophan catabolic process	4.72E−04
	Tryptophan metabolic process	5.11E−04
	Positive regulation of transcription, DNA-templated	5.11E−04
Hypomethylation-high expression	Regulation of transforming growth factor beta2 production	1.43E−02
	Regulation of receptor recycling	6.14E−04
	Membrane raft assembly	1.43E−02
	Telencephalon cell migration	1.43E−02
	Regulation of endothelial cell chemotaxis to fibroblast growth factor	1.63E−02
	Positive regulation of vesicle fusion	1.43E−02
	Negative regulation of protein depolymerization	8.40E−04
	Positive regulation of receptor binding	1.43E−02
	Nodal signaling pathway	1.83E−02
	Positive regulation of vascular endothelial cell proliferation	2.03E−02

**Fig. 16:**
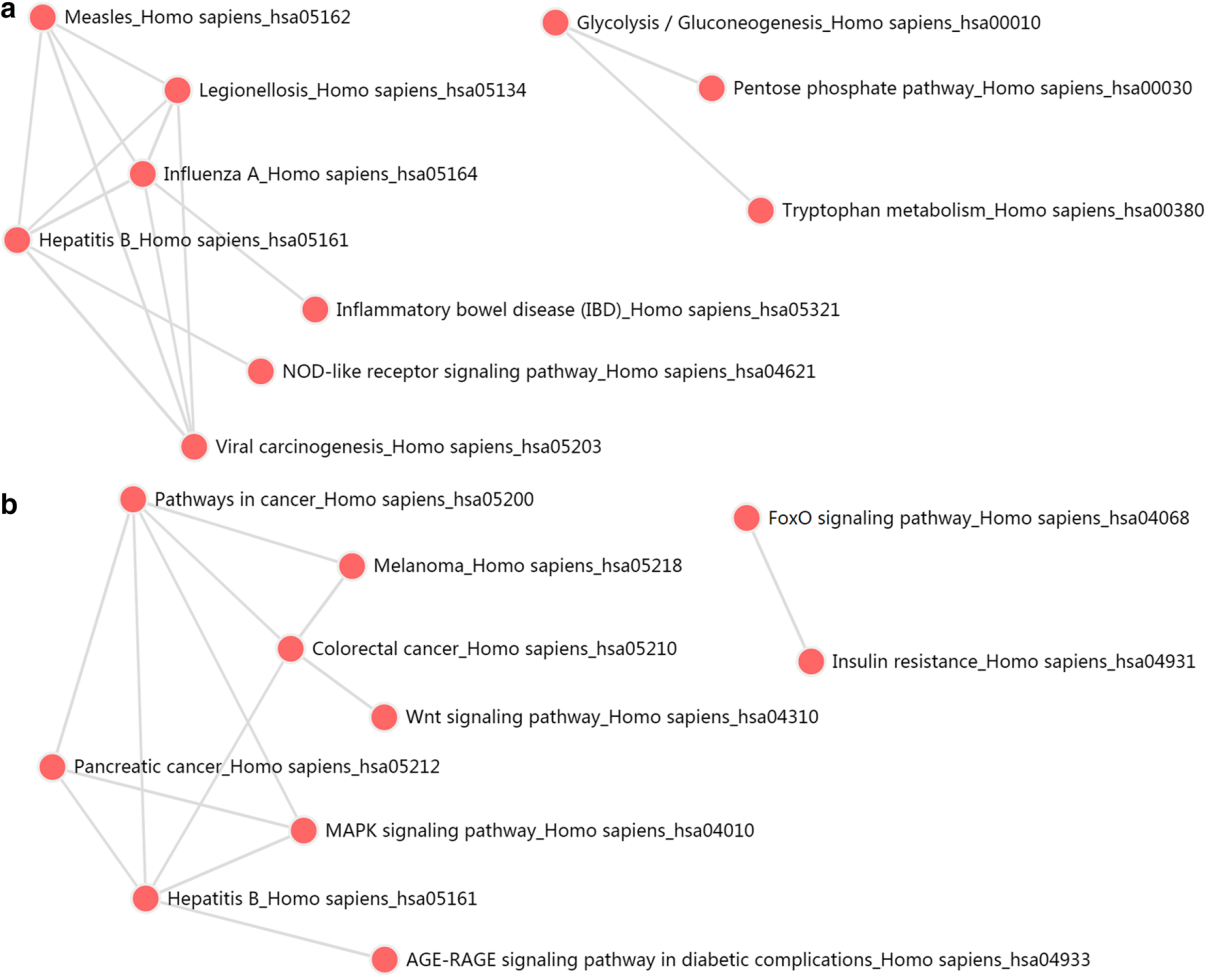
KEGG pathway analysis of aberrantly methylated-differentially expressed genes in PCa

**Fig. 17:**
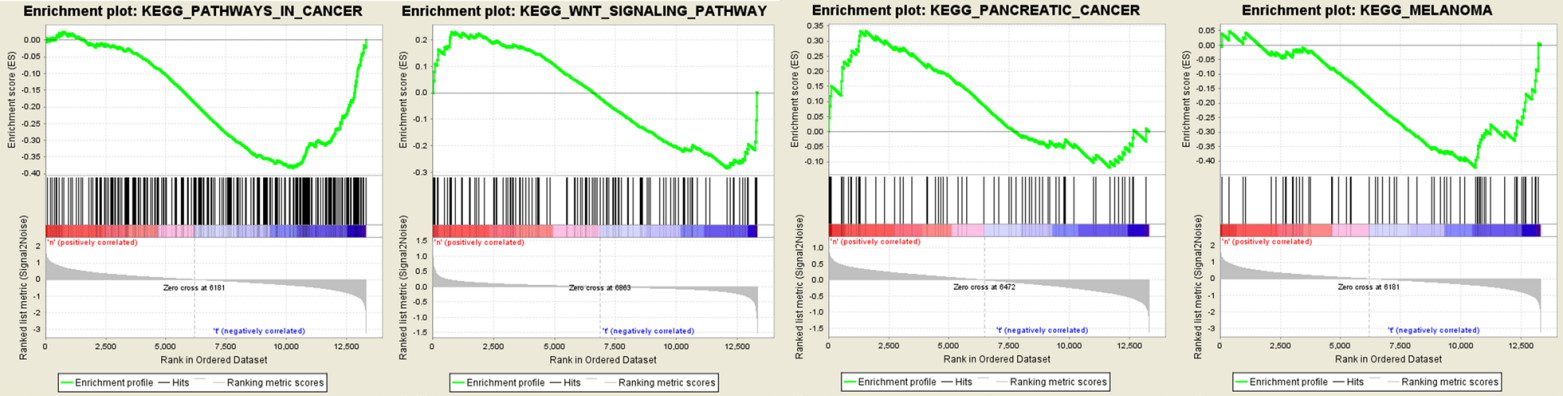
Pathway enrichment identified by GSEA of aberrantly methylated-differentially expressed genes in PCa

KEGG pathways analysis were also significantly enriched in PCa genes in Table 4. And the relationships between these pathways were shown in Fig. 16. Furthermore, GSEA (Gene Set Enrichment Analysis) was conducted to verify KEGG enrichment analysis in Fig. 16. All of these pathways were significantly enriched in Pathway in cancer, Wnt signaling pathway, Pancreatic cancer and Melanoma (Fig. 17). And most of these genes were up-regulated, which show a credible knowledge of PCa.

**Table 4:** KEGG pathway analysis of aberrantly methylated-differentially expressed genes in PCa

Category	Pathway	P-value
Hypermethylation-low expression	NOD-like receptor signaling pathway	7.37E−04
	Pentose phosphate pathway	3.64E−03
	Viral carcinogenesis	3.76E−03
	Tryptophan metabolism	6.84E−03
	Measles	8.68E−03
	Hepatitis B	1.05E−02
	Legionellosis	1.26E−02
	Cytosolic DNA-sensing pathway	1.69E−02
	Influenza A	1.71E−02
	Inflammatory bowel disease (IBD)	1.74E−02
Hypomethylation-high expression	Pathways in cancer	1.47E−04
	MAPK signaling pathway	1.67E−04
	Wnt signaling pathway	2.01E−04
	Colorectal cancer	2.78E−04
	Pancreatic cancer	3.35E−04
	Melanoma	4.15E−04
	AGE-RAGE signaling pathway in diabetic complications	1.16E−03
	Insulin resistance	1.44E−03
	FoxO signaling pathway	2.55E−03
	Hepatitis B	3.32E−03

### PPI network construction and module analysis

**Fig. 18:**
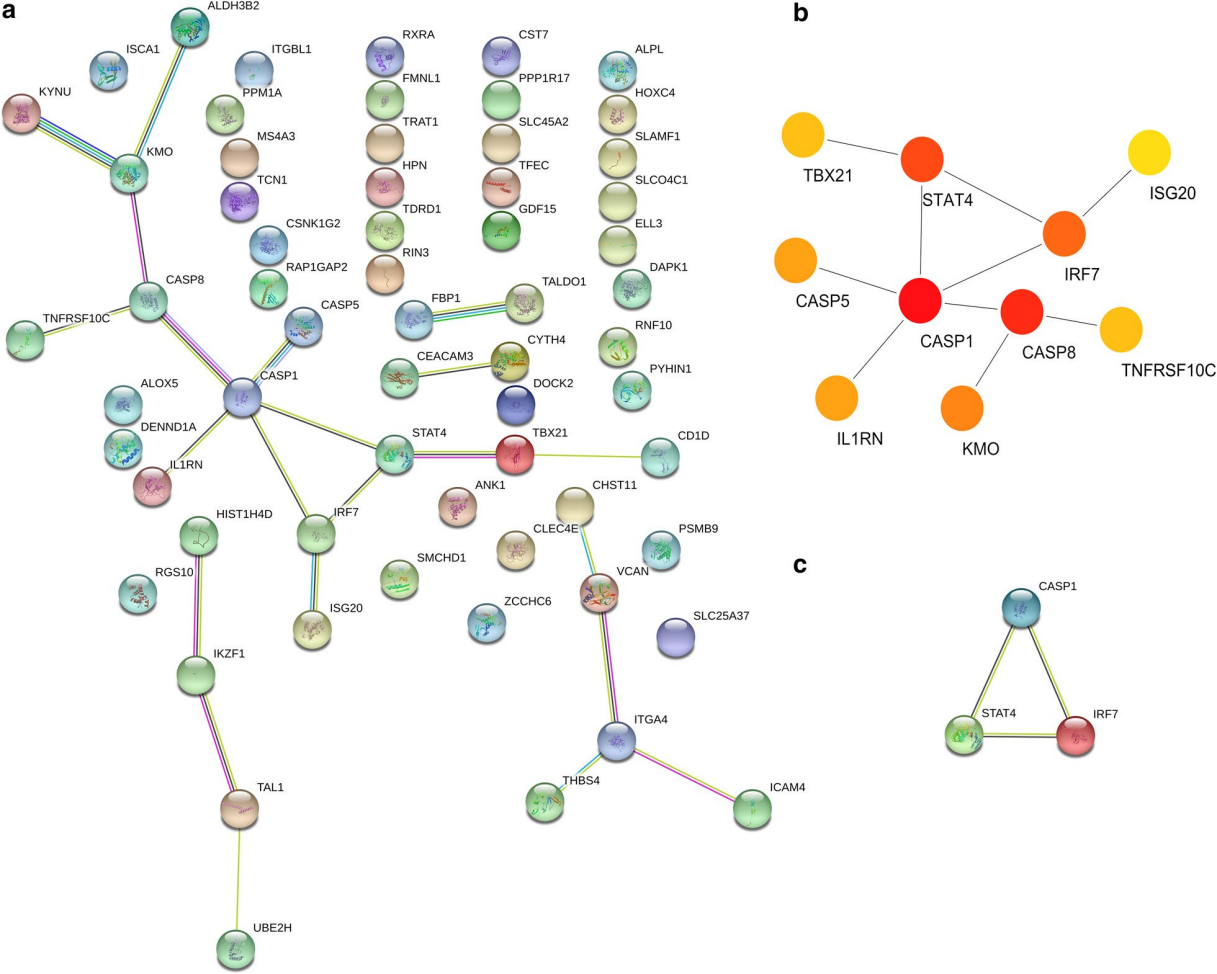
PPI network and hypermethylation-low expression genes (**a** PPI network; **b** top module; c hub genes)

All hypermethylation-low expressed genes and hypomethylation-high expressed genes were uploaded to the STRING database for constructing PPI network. Moreover, all these nodes and edges were analyzed using Cytoscape plug-ins called cytoHubba. For hypermethylation-low expressed genes, PPI network was shown in Fig. 18a and top modules were displayed in Fig. 18b. Hub genes were CASP1, STAT4 and IRF7.

**Fig. 19:**
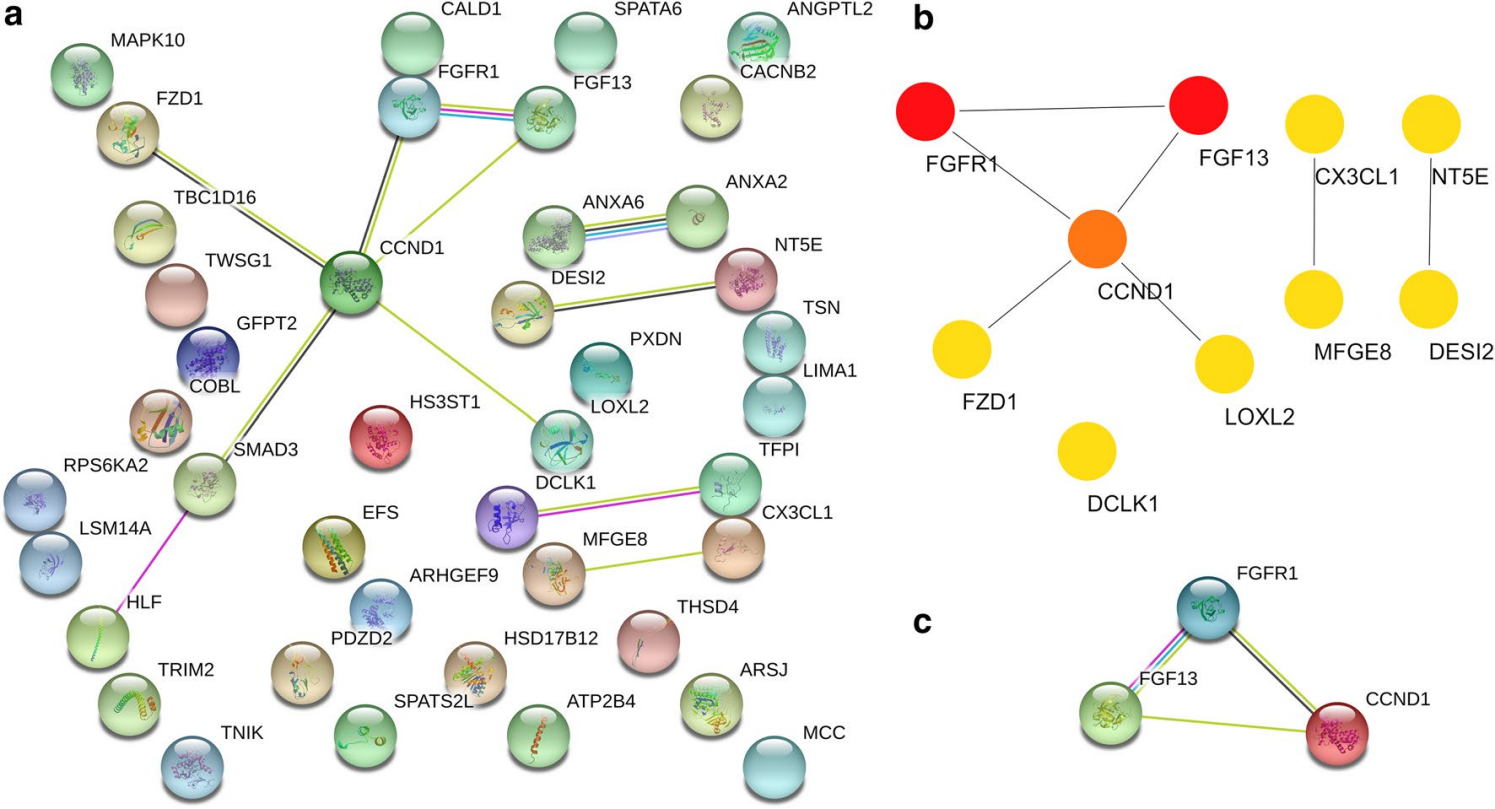
PPI network and hypomethylation-high expression genes (**a** PPI network; **b** top module; **c** hub genes)

For hypomethylation-high expressed genes, PPI network was shown in Fig. 19a and top modules were displayed in Fig. 19b. Hub genes were FGFR1, FGF13 and CCND1. Biological process of significant hub genes were demonstrated in Table 5.

**Table 5:** GO terms analysis of hub genes in PCa

Category	Term	P-value
Hypermethylation-low expression	Negative regulation of myeloid cell apoptotic process	1.05E−03
	Positive regulation of tumor necrosis factor-mediated signaling pathway	1.20E−03
	Regulation of macrophage apoptotic process	1.05E−03
	Regulation of myd88-independent toll-like receptor signaling pathway	1.35E−03
	Protein autoprocessing	1.50E−03
	Establishment of viral latency	1.50E−03
	Interleukin-23-mediated signaling pathway	1.50E−03
	Cellular response to interferon-gamma	1.01E−04
	Interleukin-21-mediated signaling pathway	1.35E−03
	Regulation of myeloid leukocyte differentiation	1.65E−03
Hypomethylation-high expression	Telencephalon cell migration	1.05E−03
	Regulation of endothelial cell chemotaxis to fibroblast growth factor	1.20E−03
	Positive regulation of vascular endothelial cell proliferation	1.50E−03
	Response to UV-A	1.05E−03
	Cerebral cortex cell migration	1.05E−03
	Regulation of collateral sprouting	1.20E−03
	Neuron migration	1.23E−05
	Positive regulation of protein serine/threonine kinase activity	1.05E−04
	Mitotic G1 DNA damage checkpoint	1.65E−03
	Regulation of cardiac muscle cell action potential involved in regulation of contraction	1.35E−03

### Interlink between candidate genes with prostate specific antigen (PSA)

**Fig. 20:**
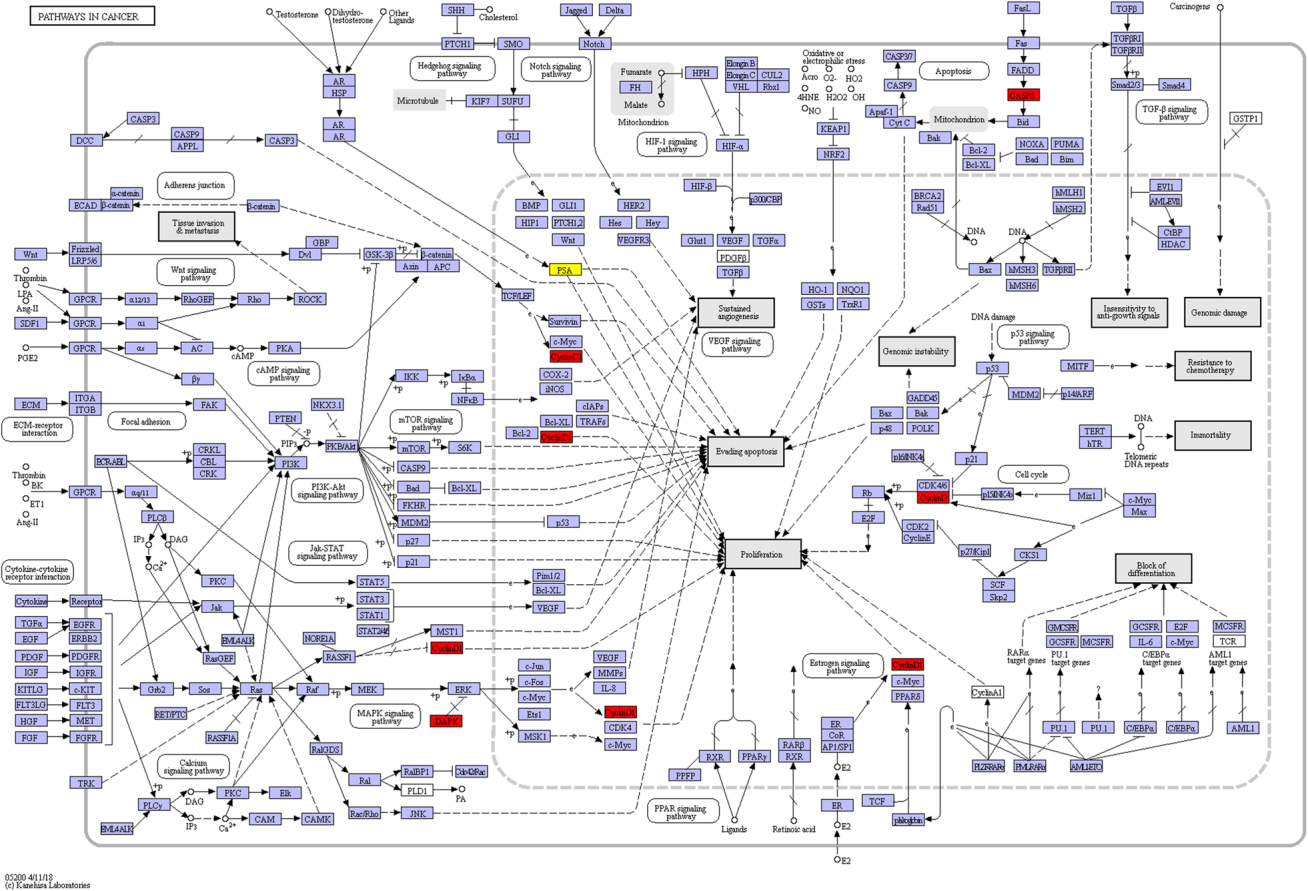
Pathways for candidate genes and PSA (red: candidate genes; yellow: PSA)

Prostate specific antigen (PAS) as a biomarker was discovered for diagnosing being and malignant prostate disease in 1960 [34]. Later, cumulative research has uncovered the antigens in the prostate and semen [35–43]. Prostate specific antigen is a protein expressed by multiple non-prostatic tissues in men and women. To obtain the relationship between candidate genes with PSA, a combined bioinformatics tools were utilized. Firstly, the Universal Protein Resource (UniProt) was used to get the PAS amino acid sequence (Additional file 1). Secondly, the PAS amino acid sequence and candidate genes were uploaded to BlastKOALA (https://www.kegg.jp/blastkoala/) for genome annotation. Finally, all the annotated genes were uploaded to KEGG PATHWAY Database (https://www.kegg.jp/kegg/pathway.html) for mapping pathway in Fig. 20. CASP8, CCND1, DAPK1 and PSA are involved in pathways in cancer. CASP8 (Caspase-8) paly essential role in apoptosis [44]. Apoptosis is regulated by CASP8 [45]. Some clinical study has confirmed that overexpression of CCND1 (Cyclin D1) is a common biomarker for treatment [46] and being ignored to cisplatin resistance in prostate cancer [47, 48]. Death associated protein kinase 1 (DAPK1) plays a critical role in apoptosis. The methylation of DAPK1 has interlink with cancer.

## Discussion

Since microarray and high-throughput sequencing can provide expression levels of thousands of genes in human genome simultaneously, it has been widely used to predict the potential therapeutic targets for PCa. In this study, we conducted a combined analysis of two types of microarray chips (DNA methylation and gene expression profile datasets) of PCa for uncovering the epigenetic and genetic mechanisms in PCa using bioinformatics analysis tools. By overlapping DEGs, DMGs and TSGs (tumor suppressor genes), we identified some candidate tumor suppressor genes that can provide new ideas for diagnosis, therapy and biomarker studies in PCa. In order to better understand the molecular mechanism of candidate tumor suppressor genes and hub genes, GO, KEGG pathway and PPI analysis were further performed.

The results demonstrated that these hypermethylation-low expressed genes were enriched in cytokine-mediated signaling pathway, benzene-containing compound metabolic process, kynurenine metabolic process, positive regulation of cysteine-type endopeptidase activity involved in apoptotic process, indolalkylamine catabolic process, response to vitamin, cellular response to mechanical stimulus, tryptophan catabolic process, tryptophan metabolic process and positive regulation of transcription, DNA-templated.

This indicated that cytokines is associated with prostate cancer. Some evidence shows the role of cytokines in prostate carcinogenesis. Cytokines are widely recognized as crucial factors in cancer development. In the progress of cancer development, cytokines were released by other immune cells. And cytokines enhance therapeutic resistances through EMT activation in tumor microenvironment [49]. Kynurenine has relationship with immune escape of tumor cells [50]. Srekumar et al. found that Kynurenine was associated with prostate cancer (PCa) progression [51]. More and more experimental researches have indicated that vitamin D has the effects of anti-prostate tumor [52, 53]. Like vitamin D, vitamin C was also natural product that has the property of antioxidant [54]. So vitamin C also plays an important role for antitumor due to characterize of antioxidant [55].

Furthermore, the enriched KEGG pathways of hypermethylation-low expressed genes have significant enrichment in NOD-like receptor signaling pathway, Pentose phosphate pathway, Viral carcinogenesis, Tryptophan metabolism, Measles_Homo sapiens, Hepatitis B, Legionellosis, Cytosolic DNA-sensing pathway and Influenza A and Inflammatory bowel disease (IBD). Cumulative evidence revealed that the pentose phosphate pathway (PPP) is a metabolic pathway, parallel to glycolysis that generates NADPH, nucleotides and nucleic acids [56]. Heritable factors and environmental factors may cause prostate cancer together. Recent research focus on the role of viral infections in prostate cancer [57]. Cytosolic DNA sensing is associated to the secretion of cytokines [58]. Cytosolic DNA sensing mediates robust antimicrobial. Cancer cells often acquire genetic or epigenetic alterations [59, 60]. Cytosolic DNA sensing is important for tumor control. In cytosolic DNA sensing, transcriptional and post-translational signaling modules enable the release of immunomodulatory cytokines [58].

After constructing PPI network for hypomethylation-high expression genes, a novel Cytoscape plugin called cytoHubba was introduced for ranking nodes in a network by the network. CytoHubba provides 12 topological analysis methods including Betweenness, BottleNeck Closeness, Clustering Coefficient, Degree, DMNC, EcCentricity, EPC, MCC, MNC, Radiality and Stress [23]. The hub genes appeared to be FGFR1, FGF13 AND CCND1.

Type 1 fibroblast growth factor receptor binding (FGFR1) was an important factor in tumor initiation and progression in prostate cancer. The activation of FGFR1 regulate the EMT in cancer progression [61]. Amplification of FGFR1 has been well studied [62, 63]. And epithelial mesenchymal-transition (EMT) may play a crucial role during in tumor metastasis and progression [64]. Recent studies have indicated that inducing EMT can affect tumor microenvironment [65].

Fibroblast growth factor 13 (FGF13) is overexpressed in several types of cancer [66, 67]. FGF13 inhibit ribosomal RNA synthesis, and may be an interplay with p53 involving a nucleolus-dependent mechanism [68]. It is possible that FGF13 may similarly signal to p53 by the same underlying molecular mechanism. FGF13 expression would activate p53, whereas the up-regulation of miR-504 would antagonize such an effect. It will be important to explore this issue directly in future studies [69]. Although the augmented FGF13 expression in tumors is unlikely to be a cancer driver, it is not merely a passenger, because it allows the cancer cells to cope with undesirable side effects of oncogene activation [68]. So FGF 13 may be reviewed as a cancer switch [70]. Cycline D1 (CCND1) plays a significant role in cell cycle. The over-expression of CCND1 in human tumors has been indicated as proto-oncogenes [71–77].

For tumor suppressor genes, IKAROS family zinc finger 1 (IKZF1) is a key regulator factor that enhanced immune infiltrate recruitment and tumor sensitivity in several tumors. Overexpression of IKZF1 can activate autoimmune susceptibility via infiltrating NKG2D^+^, CD8^+^ T cells [78]. Protein phosphatase, Mg2+/Mn2+ dependent 1A (PPM1A) is a phosphatase that has been the function of dephosphorylating TGF-β–activated P-Smad2/3, p38 and regulating several tumor-related signaling pathways [79, 80]. As a phosphatase PPM1A plays a significant role in cell cycle progression, cell proliferation, and apoptosis [81–83]. Previous study indicated that metastatic prostate cancer had lower PPM1A expression compared with primary tumor [84]. Overexpression of PPM1A has been reported to activate the expression of tumor suppressor gene TP53 [82] and increased PPM1A expression inhibited the activity of NF-kB in promoting prostate cancer invasion and metastasis [85]. Fructose-bisphosphatase 1 (FBP1) palys negative regulation roles in glycolysis and affects some process of survival, proliferation and metastasis in tumor cells [86, 87]. Some studies also showed that overexpressed FBP1 in prostate cancers can be as a tumor biomarker [88, 89]. Structural maintenance of chromosomes flexible hinge domain containing 1 (SMCHD1) is a chromatin protein associated with epigenetic modifier [90]. Alkaline phosphatase (ALPL) is a non-specific hydrolase and plays crucial role in regulating phosphate metabolism involved in cell cycle, growth, apoptosis and signal transduction pathways [91]. The abnormal expression of ALP can be a biomarker for prostate cancer [92]. The expression of caspase-5 (CACP5) is very low in many normal tissues, while its expression plays an important role in cell apoptosis [93]. Pyrin and HIN domain family member 1 (PYHIN1) has the function of tumor suppression [94]. Death associated protein kinase 1 (DAPK1) is a kinase regulated neuronal apoptosis by calcium-calmodulin. So far, a large number of studies have demonstrated that DAPK1 acts as a positive mediator interact with several proteins, such as DAPK1-p53 signaling, DAPK1-tau signaling, and DAPK1-DANGER signaling [95–98]. Caspase-8 (CASP8) acts as an environment sensor in the control of cell death. CASP8 induced a wide range of biological process, such as the control of apoptosis and necroptosis [99].

All the genes and pathways in this investigation are all based on bioinformatics methods. So no clinical samples were used to validate the data. But a shot literature review was introduced to confirm out findings.

## Conclusions

In this study, a joint bioinformatics analysis method was used for indicating candidate tumor suppressor genes and pathways in PCa by combined gene methylation microarrays and gene expression microarray, this may provide a set of useful targets for future investigation into the molecular mechanisms and biomarkers. Candidate tumor suppressor genes were IKZF1, PPM1A, FBP1, SMCHD1, ALPL, CASP5, PYHIN1, DAPK1 and CASP8. Hub genes were FGFR1, FGF13 and CCND1 that may contribute to the finding of molecular mechanisms underlying the initiation and development of PCa. Meanwhile, there were some limitations should be declared in this work. The clinical gene methylation profiling and gene expression profiling datasets were not analyzed in this study. Besides, the hub genes on gene expression were only validated in TCGA database.

## Additional file

**Additional file 1.** Supplementary S1-PAS protein sequence.

## Abbreviations

PCa: prostate cancer
GEO: Gene Expression Omnibus
DEG: differentially expressed gene
DMG: differentially methylated gene
TSG: tumor suppressor gene
DAVID: Database for Annotation, Visualization and Integrated Discovery
GO: gene ontology
KEGG: Kyoto Encyclopedia of Genes and Genomes
PPI: protein–protein interaction
STRING: Search Tool for the Retrieval of Interacting Genes
TCGA: The Cancer Genome Atlas
BP: biological process
BH: Benjamini and Hochberg
log_2_FC: log_2_-fold change
